# Dietary Methionine Restriction Alleviates Choline-Induced Tri-Methylamine-N-Oxide (TMAO) Elevation by Manipulating Gut Microbiota in Mice

**DOI:** 10.3390/nu15010206

**Published:** 2023-01-01

**Authors:** Manman Lu, Yuhui Yang, Yuncong Xu, Xiaoyue Wang, Bo Li, Guowei Le, Yanli Xie

**Affiliations:** 1 National Engineering Laboratory/Key Laboratory of Henan Province, College of Food Science and Engineering, Henan University of Technology, Zhengzhou 450001, China; 2 Beijing Advanced Innovation Center for Food Nutrition and Human Health, College of Food Science and Nutritional Engineering, China Agricultural University, Beijing 100083, China; 3 State Key Laboratory of Food Science and Technology, Jiangnan University, Wuxi 214122, China

**Keywords:** methionine restriction, high choline, trimethylamine, trimethylamine-N-oxide, gut microbiota, trimethylamine lyase

## Abstract

Dietary methionine restriction (MR) has been shown to decrease plasma trimethylamine-N-oxide (TMAO) levels in high-fat diet mice; however, the specific mechanism used is unknown. We speculated that the underlying mechanism is related with the gut microbiota, and this study aimed to confirm the hypothesis. In this study, we initially carried out an in vitro fermentation experiment and found that MR could reduce the ability of gut microbiota found in the contents of healthy mice and the feces of healthy humans to produce trimethylamine (TMA). Subsequently, mice were fed a normal diet (CON, 0.20% choline + 0.86% methionine), high-choline diet (H-CHO, 1.20% choline + 0.86% methionine), or high-choline + methionine-restricted diet (H-CHO+MR, 1.20% choline + 0.17% methionine) for 3 months. Our results revealed that MR decreased plasma TMA and TMAO levels in H-CHO-diet-fed mice without changing hepatic FMO3 gene expression and enzyme activity, significantly decreased TMA levels and expression of choline TMA-lyase (*CutC*) and its activator *CutD*, and decreased CutC activity in the intestine. Moreover, MR significantly decreased the abundance of TMA-producing bacteria, including *Escherichia-Shigella* (*Proteobacteria* phylum) and *Anaerococcus* (*Firmicutes* phylum), and significantly increased the abundance of short-chain fatty acid (SCFA)-producing bacteria and SCFA levels. Furthermore, both MR and sodium butyrate supplementation significantly inhibited bacterial growth, down-regulated *CutC* gene expression levels in TMA-producing bacteria, including *Escherichia fergusonii ATCC 35469* and *Anaerococcus hydrogenalis DSM 7454* and decreased TMA production from bacterial growth under in vitro anaerobic fermentation conditions. In conclusion, dietary MR alleviates choline-induced TMAO elevation by manipulating gut microbiota in mice and may be a promising approach to reducing circulating TMAO levels and TMAO-induced atherosclerosis.

## 1. Introduction

Trimethylamine-N-oxide (TMAO) is oxidized from trimethylamine (TMA), a biologically active molecule, which is a metabolite yielded from the gut microbial degradation of choline, betaine, and L-carnitine [1,2] and transformed into TMAO in the liver through the action of flavin mono-oxygenase (FMO) family members. FMO3 contributes ≥ 90% of the total hepatic activity involving oxidation of TMA to TMAO [3]. Foods rich in TMA and its precursors, such as red meat, eggs, poultry, milk, marine fish, and beets, are principal dietary sources for TMAO production [1,4]. TMAO appears to affect lipid metabolism and contributes to the progression of atherosclerosis (AS) by causing cholesterol accumulation in cells. It can also directly affect platelet function and promote an inflammatory response [3,5]. Dietary supplementation with TMAO increases the level of circulating TMAO and propels up-regulation of multiple macrophage scavenger receptors related to AS [6,7]. Thus, circulating TMAO levels are highly associated with AS and TMAO levels explain 11% of the changes in AS [3]. Numerous clinic-based studies further identified the positive association between TMAO and AS [6,8,9]. The absence of normal production of urinary TMA in germ-free mice revealed that intestinal microflora is required in TMAO formation from dietary precursors [10], and antibiotics can restrain plasma TMAO concentrations in mice [6] and in humans [8]. Previous studies also demonstrate that the intestinal microflora structure is correlated with circulating TMAO levels [11,12,13] for TMA is generated by gut microbiota using three kinds of enzyme complexes: *CutC*/*D* (choline TMA-lyase gene and its activator) encode enzymes for anaerobic degradation of choline to TMA; *CntA*/*B* encode a two-subunit Rieske-type oxygenase/reductase for carnitine conversion to TMA; *YeaW*/*X* encode a two-subunit Rieske-type oxygenase/reductase for betaine conversion to TMA [1,14,15,16]. Hence, the gut microbiota was proposed as a latent target to regulate TMA and TMAO generation.

Since plasma TMAO levels are highly correlated with dietary patterns and the nutritional composition of foods [17,18], dietary intervention may be an alternative strategy for inhibiting TMAO production [19]. A methionine-restricted (MR) diet has emerged as a highly preferable dietary intervention strategy since it is reported to exert many beneficial effects including the promotion of vascular health [20], reduction in fat deposition [21], improvement of glucose homeostasis [22], improving oxidative stress and inflammation [23,24], and a prolonged life span [25]. Our previous studies found that a MR diet can reduce plasma TMA and TMAO levels in both high-fat-diet (HFD) and normal-diet-fed mice [26,27]. In addition, the MR diet is able to reduce the abundance of *Escherichia-Shigella* while increasing the abundance of short-chain fatty acid (SCFA)-producing bacteria in different-fat-diet-fed mice [24,28,29]. *Escherichia-Shigella* has been found to contain *CutC*/*D*, *CntA*/*B*, and *YeaW*/*X* [14,30]. Inhibiting TMA-lyases by altering gut microbiota composition is a promising strategy to reduce TMAO formation [16,31]. Nevertheless, the influences of MR on the expression of *CutC*/*D*, *CntA*/*B*, and *YeaW*/*X* in gut microbiota remain unknown. SCFAs, especially butyrate, were reported to reduce circulating TMA levels and inhibit the HFD-induced AS [32]. However, whether MR can reduce TMA and TMAO levels in high-choline (H-CHO)-diet-fed mice and whether its possible mechanisms is related to gut microbiota remain obscure.

Therefore, we proposed a hypothesis that MR may reduce the circulation levels of TMAO by manipulating gut microbiota. Here, bacteria isolated from the intestines of healthy mice and humans were cultured in vitro under MR and anaerobic fermentation conditions, and TMA levels and TMA-lyase expression levels were examined. In the animal experiment, we examined the influences of MR on circulating TMA and TMAO levels, hepatic FMO3 gene expression levels and enzyme activity, the AS index, the gut microbiota composition, intestinal TMA levels, and the expression of *CutC*/*CutD* as well as CutC activity in cecal and colonic contents of H-CHO-diet-fed mice. Furthermore, the influences of MR and sodium butyrate supplementation, respectively, on bacterial growth, TMA production by selected bacteria, and *CutC* expression and activity in the bacteria under anaerobic fermentation conditions were explored.

## 2. Materials and Methods

### 2.1. Experimental Design

#### 2.1.1. Experiment 1: Effects of Different Levels of MR on TMA Production by Bacteria from Colonic Contents of Healthy Mice and Feces from Healthy Humans

The colonic contents of healthy mice and feces from healthy humans (three men and three women, no use of antibiotics in the past three months, provide voluntarily) were collected. The samples (containing 0.2% choline but with different levels of methionine: CON, 0.86% methionine; MR-20%, 0.69% methionine; MR-40%, 0.52% methionine; MR-60%, 0.34% methionine; MR-80%, 0.17% methionine) were diluted with water in a 1:20 ratio to a choline concentration of 100 μg/mL and fermented with the colonic contents and feces (anaerobic condition: 10% CO_2_-10% H_2_-80% N_2_) for 12 h.

#### 2.1.2. Experiment 2: Effects of MR on Circulating TMA/TMAO Levels and Gut Microbiota Composition in Mice

The animal experiment was ratified by the Animal Care and Use Committee at Jiangnan University, and all the experimental manipulations were undertaken in accordance with the National Guidelines for Experimental Animal Welfare (MOST of PR China, 2006). A total of 30 eight-week-old, male Institute of Cancer Research (ICR) mice (Shanghai Slac Laboratory animal Co., Ltd., Shanghai, China) were kept in the SPF-grade laboratory for 1 week to adapt to laboratory conditions of a 12:12 h (T = 24) light dark cycle, constant temperature of 22 ± 1 °C, relative humidity of 60%, and food and water ad libitum. After one week of adaptive feeding, all experimental mice were randomly divided into three groups (*n* = 10): (1) CON, mice fed a normal diet (0.20% choline + 0.86% methionine); (2) H-CHO, mice fed a high-choline diet (1.20% choline + 0.86% methionine); (3) H-CHO+MR, mice fed a high-choline + methionine-restricted diet (1.20% choline + 0.17% methionine). Methionine dose [33,34] and choline dose [7,35] used in this study were according to those used in previous studies. The dietary formulations are displayed in Table S1, and the experiment lasted for 90 days.

Feces were collected for 3 consecutive days in sterile conditions according to a previously described method, with appreciate modifications [36]. Then, all the experimental mice were anesthetized by an intraperitoneal injection of sodium pentobarbital. Blood samples were collected from the eyeball into a heparin sodium anticoagulant tube and were centrifuged (4 °C, 4000 rpm, 10 min) after placed for 30 min, and the plasma samples were then collected and stored at −80 °C. Afterwards, the mice were executed and their liver, aorta, cecum, and colon tissues were removed on ice. Cecal and colonic contents were separated from the cecum and colon, respectively, and stored at −80 °C until used. The colon tissues were fixed by immersion in 4% neutral formaldehyde solution and stored at 4 °C until used for hematoxylin and eosin (H&E) staining.

#### 2.1.3. Experiment 3: Effects of MR/Sodium Butyrate Supplement on TMA Production from *Escherichia fergusonii* and *Anaerococcus hydrogenalis*

*Escherichia fergusonii ATCC 35469* and *Anaerococcus hydrogenalis DSM 7454* purchased from China General Microbiological Culture Collection Center (CGMCC) and bacteria from human fecal samples were cultured in CON media or MR-80%/sodium butyrate supplement media at 37 °C (anaerobic condition: 10% CO_2_-10% H_2_-80% N_2_) using the AW400SG anaerobic workstation (Electrotek, West Yorkshire, UK). The colony numbers were assessed using an ultraviolet spectro-photometer (UV-1800, Shimadzu, Kyoto, Japan) set to 600 nm. TMA production by *Escherichia fergusonii ATCC 35469* and *Anaerococcus hydrogenalis DSM 7454*, and bacteria from human feces samples was quantified as described previously [37].

### 2.2. Detection of TMA/TMAO Levels

The TMA levels in mouse colonic contents, those in human fecal fermentation broth, plasma and colonic/cecal contents, the TMA production by *Escherichia fergusonii ATCC 35469*, *Anaerococcus hydrogenalis DSM 7454*, and the gut microbiota from human feces samples, and TMAO levels in the plasma were detected using a previously described method [32]. High-performance liquid chromatography tandem mass spectrometry (HPLC-MS/MS) was performed using a mass spectrometer (Thermo Fisher Scientific, San Jose, CA, USA).

### 2.3. Detection of AS-Related Indicators

High-density lipoprotein cholesterol (HDL-C) and total cholesterol levels in the plasma were detected using assay kits (ShangHai FengHui medical science and technology Co., Ltd., Shanghai, China). Then, the AS index was calculated. AS index = (total cholesterol − HDL-C)/HDL-C [38].

### 2.4. Quantitative Real-Time Polymerase Chain Reaction (qRT-PCR)

Samples were pre-processed as previously described [39], and total bacterial DNA in gut microbiota from mice colonic contents and human feces fermentation broth, mice colonic contents, mice cecal contents, mice feces, *Escherichia fergusonii ATCC 35469*, and *Anaerococcus hydrogenalis DSM 7454* was extracted using an Ezup Column Bacteria Genomic DNA Purification kit (Sangon Biotech Co., Ltd., Shanghai, China). The obtained genomic DNA samples were further processed according to our previous method for subsequent analysis [40]. The total RNA of the liver, aorta, and colon of mice was extracted using Trizol reagent (Vazyme Biotech Co., Ltd., Nanjing, China), and subsequent operation procedures were carried out according to our previous research [40]. Specific gene primers were designed (Table S2). The relative expression of each gene was calculated using the method of 2^−ΔΔCT^ [41].

### 2.5. Assays for Activity of FMO3/CutC

The activity of FMO3 in the liver was examined as previously reported in the literature [3]. Briefly, mouse liver tissue was homogenized in RIPA lysate containing 1 mmol/L phenylmethanesulfonyl fluoride (PMSF), and the protein content was determined using a BCA protein quantification kit (Beyotime Biotechnology, Shanghai, China). Then, 1.0 mg of liver protein was added to d9-TMA to a concentration of 100 μmol/L and mixed evenly with 100 mmol/L of nicotinamide adenine dinucleotide phosphate (NADPH). Finally, 10 mmol/L 4-hydroxypiperazine ethylene sulfonic acid (pH = 7.4) buffer was added to obtain the 250 μL reaction system. After incubating at 37 °C for 8 h, terminated the reaction by adding 0.2 mol/L formic acid. Then, the filtered samples (ValueLab filter PTFE-Q 0.2 μm, Agilent Technologies Inc., Palo Alto, CA, USA) were transferred to the sample vial, stored in the −80 °C refrigerator, and the content of the generated d9-TMAO was detected by HPLC-MS/MS. The activity of CutC in mouse colonic/cecal contents and selected bacteria (*Escherichia fergusonii* and *Anaerococcus hydrogenalis*) was measured using an assay kit (Jingmei Biotechnology Co., Ltd. Jiangsu, China).

### 2.6. Structural Analysis of Fecal Microbiota

The structural analysis of fecal microbiota was entrusted to GENEWIZ, Inc, Suzhou, China; high throughput 16S rDNA sequencing was applied to analyze the relative abundance of fecal bacteria. For more details, please refer to our previous study [40].

### 2.7. Histological Analysis of Colon Tissues

The fixed colon tissue sections were dyed with H&E using kits (Nanjing Jiancheng Bioengineering Institute, Nanjing, China). H&E-stained sections were observed under inverted fluorescence microscopy (Olympus Corporation, Tokyo, Japan) and images were acquired (400×).

### 2.8. Detection of SCFAs

The acetate, propionate, and butyrate levels in mouse cecal contents were determined using a gas chromatography coupled mass spectrometer (GC-MS-QP2010, Shimadzu, Japan) according to the published literature [42]. Total acid was calculated as the summation of acetate, propionate, and butyrate.

### 2.9. Statistical Analysis

Data were analyzed using the SPSS 17.0 and are presented as the mean ± standard error of the mean (SEM). One-way analysis of variance (ANOVA) was applied to compare the differences between the groups. Tukey’s HSD was conducted when the error terms were assumed to have equal variance, whereas Tamhane’s T2 test was applied when equal variance was not assumed. Data were considered statistically significant for *p* < 0.05 and highly significant for *p* < 0.01.

## 3. Results

### 3.1. MR Inhibited the TMA Production by the Intestinal Bacteria from Healthy Mice and Humans

**Figure 1:**
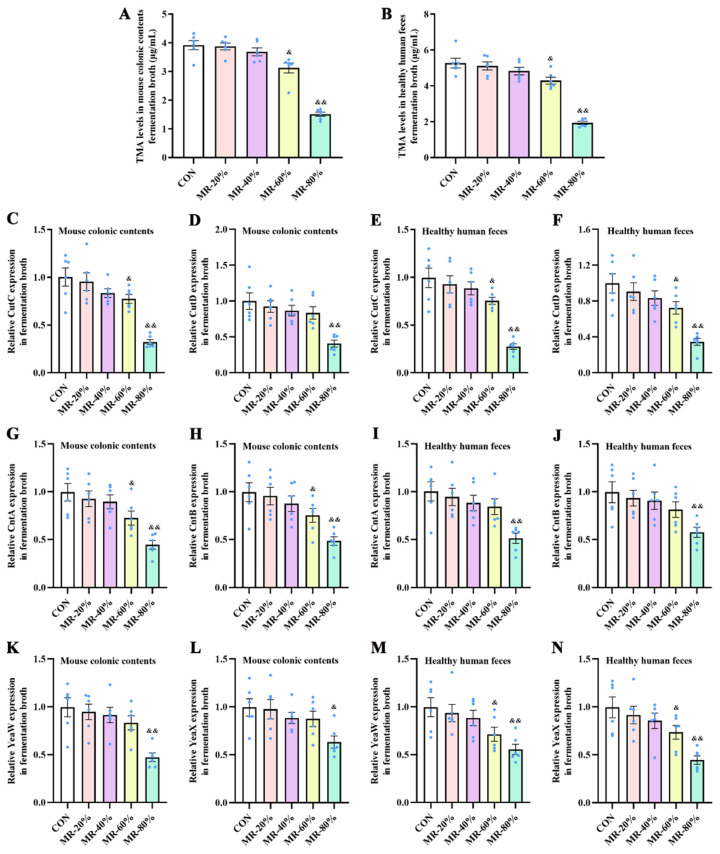
Effects of different levels of MR on TMA production by bacteria from mouse colonic contents (**A**) and healthy human feces (**B**) cultured anaerobically in fermentation medium, and on expression levels of *CutC* (**C**,**E**), *CutD* (**D**,**F**), *CntA* (**G**,**I**), *CntB* (**H**,**J**), *YeaW* (**K**,**M**), and *YeaX* (**L**,**N**) in bacteria from mouse colonic contents and healthy human feces cultured anaerobically in fermentation medium. TMA, trimethylamine; MR, methionine restriction; CON, 0.86% methionine; MR-20%, 0.69% methionine; MR-40%, 0.52% methionine; MR-60%, 0.34% methionine; MR-80%, 0.17% methionine. ^&^ *p* < 0.05, ^&&^ *p* < 0.01, compared with the CON group.

We initially tested the effects of MR in an in vitro fermentation experiment, which was first performed for preliminary investigation on the inhibitory effects of MR on TMA production by intestinal bacteria. Compared with the CON group, MR-60% significantly decreased TMA levels (Figure 1A,B) and down-regulated *CutC* expression levels (Figure 1C,E) both in mouse colonic contents and in the healthy human feces fermentation broth, significantly decreased *CutD* (Figure 1F), *YeaW* (Figure 1M), and *YeaX* (Figure 1N) expression levels in healthy human feces fermentation broth and decreased *CntA* (Figure 1G) and *CntB* (Figure 1H) expression levels in mouse colonic contents (*p* < 0.05); MR-80% highly significantly decreased TMA levels and down-regulated *CutC*, *CutD* (Figure 1D), *CntA* (Figure 1I), *CntB* (Figure 1J), and *YeaW* (Figure 1K) expression levels both in mouse colonic contents and healthy human feces fermentation broth (*p* < 0.01), significantly decreased *YeaX* (Figure 1L) expression levels in mouse colonic contents (*p* < 0.05) and highly notably reduced *YeaX* expression in healthy human feces fermentation broth (*p* < 0.01).

### 3.2. MR Decreased Plasma TMA and TMAO Levels, Body Weight, and AS Index in H-CHO-Diet Fed-Mice

**Figure 2:**
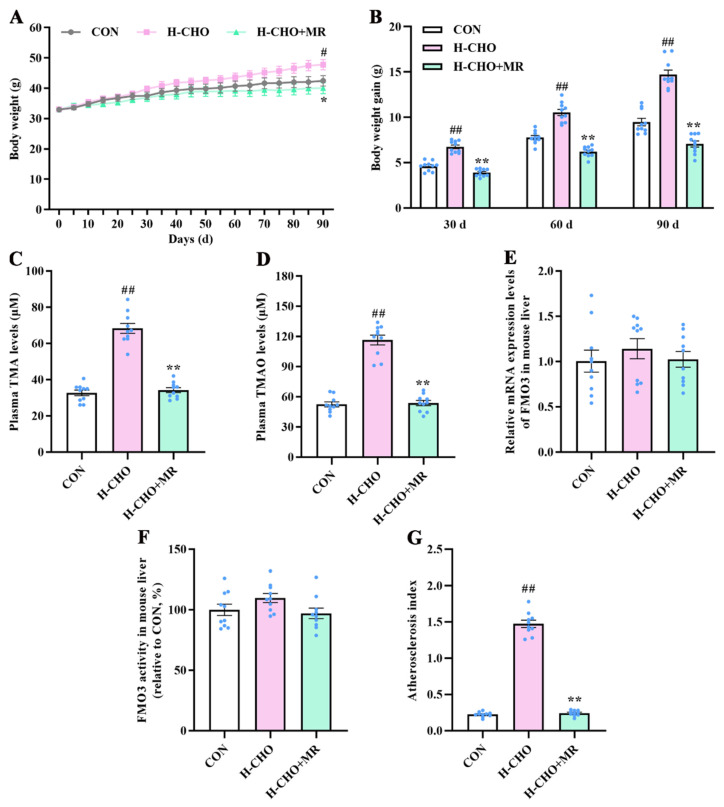
Effects of MR on body weight (**A**), body weight gain (**B**), plasma TMA (**C**) and TMAO levels (**D**), hepatic FMO3 gene expression levels (**E**) and activity (**F**), and atherosclerosis index (**G**) in mice under H-CHO diet conditions. MR, methionine restriction; CON, normal diet group; H-CHO, high-choline diet group; H-CHO+MR, high-choline + methionine restricted diet group. ^#^ *p* < 0.05, ^##^ *p* < 0.01 (H-CHO vs. CON); * *p* < 0.05, ** *p* < 0.01 (H-CHO+MR vs. H-CHO).

Plasma TMA and TMAO levels were measured to elucidate the effects of MR on circulating TMAO levels in H-CHO-diet-fed mice. Compared with the CON mice, the H-CHO diet statistically increased the body weight (Figure 2A), body weight gain (Figure 2B), plasma TMA (Figure 2C) and TMAO (Figure 2D) levels, and AS index (Figure 2G) in mice (*p* < 0.05). Compared with H-CHO mice, the H-CHO+MR diet statistically reduced body weight, body weight gain, plasma TMA and TMAO levels, and AS index in mice (*p* < 0.05). Moreover, we observed that neither the H-CHO nor H-CHO+MR diet significantly changed hepatic *FMO3* gene expression levels and FMO3 activity in mice (*p* > 0.05).

### 3.3. MR Improved Gut Microbiota Composition in H-CHO-Diet-Fed Mice

**Figure 3:**
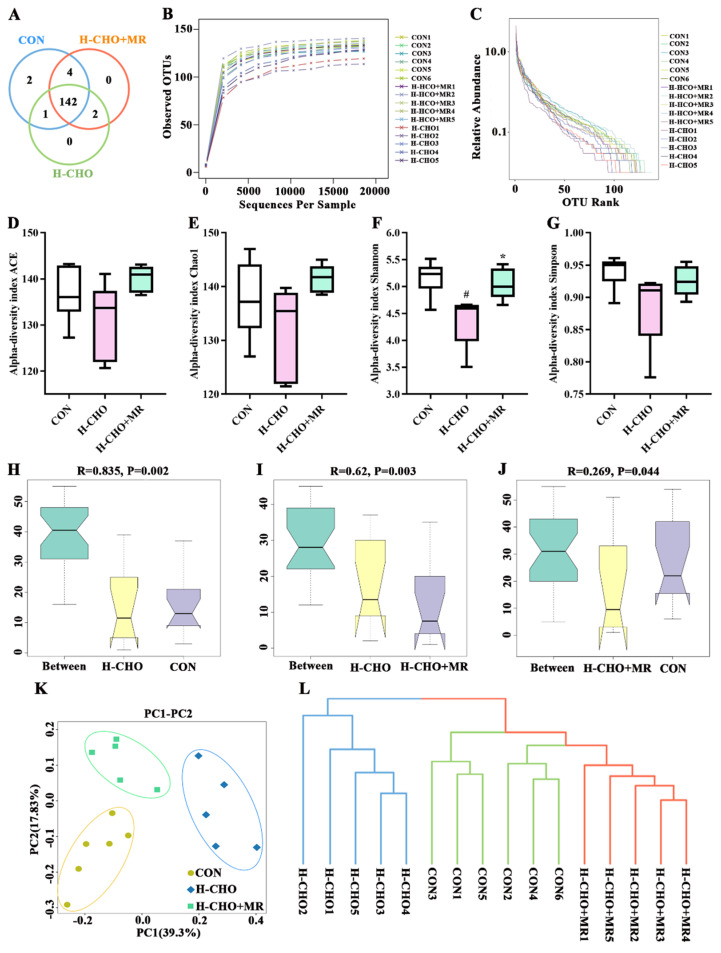
α-diversity analysis and β-diversity analysis of microbiota in mouse feces. (**A**) OTUs Venn analysis; (**B**) Rarefaction curve; (**C**) OTU Rank; (**D**) Ace index; (**E**) Chao1 index; (**F**) Shannon index; (**G**) Simpson index; (**H**) Anosim (H-CHO vs. CON), the closer the R value is to 1, the greater the difference between groups than the difference in the group, *p* < 0.05 indicates that the difference is statistically significant; (**I**) Anosim (H-CHO vs. H-CHO+MR); (**J**) Anosim (H-CHO+MR vs. CON); (**K**) PCA score plots; (**L**) unweighted unifrac cluster tree. Anosim, analysis of similarities; PCA, principal component analysis; MR, methionine restriction; OTU, operational taxonomic units; CON, normal diet group; H-CHO, high-choline diet group; H-CHO+MR, high-choline + methionine restricted diet group. ^#^ *p* < 0.05 (H-CHO vs. CON); * *p* < 0.05 (H-CHO+MR vs. H-CHO).

To explore the effects of MR on intestinal microflora in H-CHO-diet-fed mice, 16S rDNA gene high-throughput sequencing was applied to analyze gut microbiota composition. As displayed in Figure 3A, a total of 151 OTUs for all samples were generated (149 OTUs for samples from CON group, 145 OTUs for samples from H-CHO group, and 148 OTUs for samples from H-CHO+MR group). The rarefaction curves (Figure 3B) revealed that the OUT levels had no changes as the number of analyzed sequencing increased, indicating that the bacterial community was well represented. The rank-abundance curves (Figure 3C) characterize species abundance and species uniformity. Compared with the CON group, the H-CHO diet decreased species abundance and species evenness. Compared with the H-CHO group, the H-CHO+MR diet increased species abundance and species evenness. Then, the α-diversity indexes (Figure 3D–G) were calculated to evaluate the abundance and structural difference among the CON, H-CHO, and H-CHO+MR groups. Compared with CON mice, the H-CHO diet significantly decreased Shannon index in mice (*p* < 0.05). The ACE, Chao1, and Simpson indexes were also lower in H-CHO mice compared to CON mice (*p* > 0.05). Compared with H-CHO mice, the H-CHO+MR diet significantly increased Shannon index in mice (*p* < 0.05). The ACE, Chao1, and Simpson indexes were higher in mice in the H-CHO+MR group compared with mice in the H-CHO group (*p* > 0.05).

β-diversity indexes were analyzed to confirm the differences in intestinal microbial species among the CON, H-CHO, and H-CHO+MR groups of mice using analysis of similarities (Anosim, Figure 3H,I), PCA (Figure 3J) and unweighted unifrac cluster tree (Figure 3K). Anosim showed that the differences between the CON group and the H-CHO group, and between the H-CHO group and the H-CHO+MR group are greater than inter-group differences (*p* < 0.05). The species composition of the three groups could be separated clearly by PCA, and unweighted unifrac cluster tree also displayed an obviously separation.

**Figure 4:**
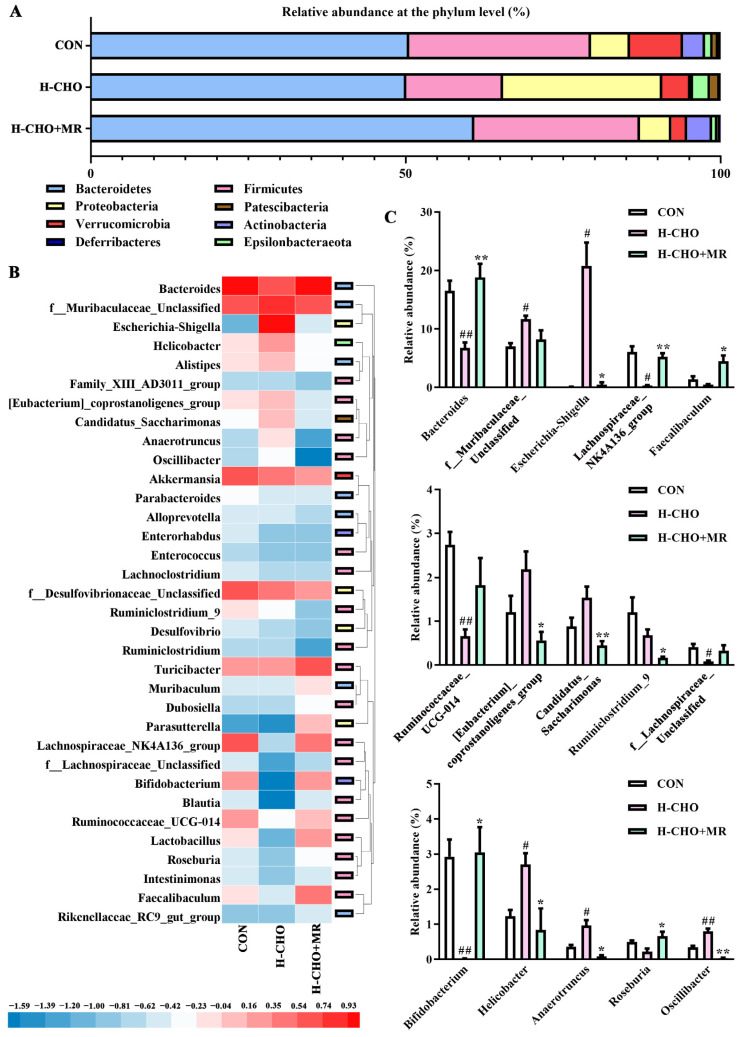
MR altered the gut microbiota composition in feces of mice fed with the H-CHO diet. (**A**) relative abundance of the major bacterial phyla in mouse feces; (**B**) heat map of the microbiota in mouse feces at the genus level (relative abundance > 0.2%); (**C**) comparison of relative abundance at the genus levels among the CON, H-CHO, and H-CHO+MR groups. MR, methionine restriction; CON, normal diet group; H-CHO, high-choline diet group; H-CHO+MR, high-choline + methionine restricted diet group. ^#^ *p* < 0.05, ^##^ *p* < 0.01 (H-CHO vs. CON); * *p* < 0.05, ** *p* < 0.01 (H-CHO+MR vs. H-CHO).

A total of 8 bacteria phyla were found in all the samples (Figure 4A). At the genus level, for clarity and visualization purposes, Figure 4B presents the most abundant taxa (those with a relative abundance of >0.2%). Further analyzes revealed 15 of the 34 bacterial genera with significance discrepancy. As displayed in Figure 4C, compared with CON mice, the H-CHO diet significantly elevated the abundance of *f__Muribaculaceae_Unclassified*, *Escherichia-Shigella*, *Helicobacter*, *Anaerotruncus*, and *Oscillibacter*, whereas significantly reduced the levels of *Bacteroidetes*, *Ruminococcaceae_UCG-014*, *Bifidobacterium, Lachnospiraceae_NK4A136_group,* and *f__Lachnospiraceae_Unclassified* in mice feces (*p* < 0.05). Compared with H-CHO mice, the H-CHO+MR diet markedly increased the abundance of *Bacteroidetes*, *Lachnospiraceae_NK4A136_group*, *Faecalibaculum*, *Bifidobacterium*, *Roseburia*, and notably reduced *Escherichia-Shigella*, *[Eubacterium]_coprostanoligenes_group*, *Ruminiclostridium_9*, *Anaerotruncus*, *Candidatus_Saccharimonas*, and *Oscillibacter* levels in mice feces (*p* < 0.05).

### 3.4. MR Decreased Intestinal TMA Levels in H-CHO-Diet-Fed Mice

**Figure 5:**
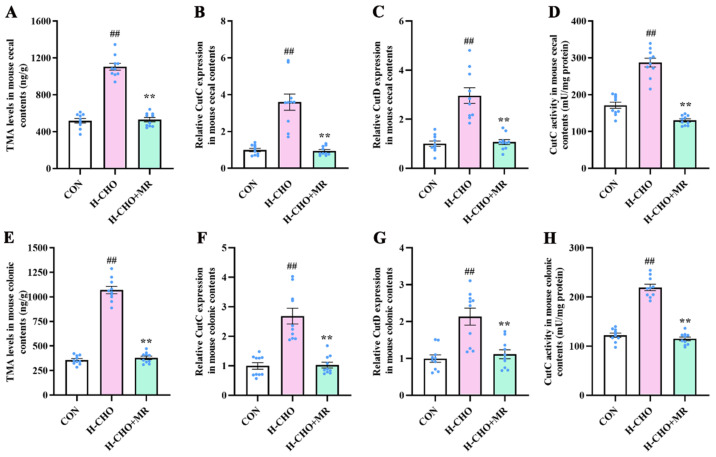
Effects of MR on TMA levels (**A**,**E**), *CutC* (**B**,**F**) and *CutD* (**C**,**G**) expression, and CutC activity (**D**,**H**) in mouse cecal contents and colonic contents under H-CHO diet conditions. MR, methionine restriction; TMA, trimethylamine; CON, normal diet group; H-CHO, high-choline diet group; H-CHO+MR, high-choline + methionine restricted diet group; CON, 0.86% methionine; MR-80%, 0.17% methionine. ^##^ *p* < 0.01 (H-CHO vs. CON); ** *p* < 0.01 (H-CHO+MR vs. H-CHO).

To further investigate the inhibitory mechanism of MR regarding plasma TMA and TMAO levels, the intestinal TMA levels were measured and the expression of the *CutC* and *CutD* as well as CutC activity in mouse cecal and colonic contents was determined. Compared with CON mice, the H-CHO diet significantly increased TMA levels in cecal contents (Figure 5A) and colonic contents (Figure 5E) of mice, and significantly increased *CutC* (Figure 5B,F) and *CutD* (Figure 5C,G) expression levels as well as CutC activity (Figure 5D,H) both in mouse cecal and colonic contents (*p* < 0.01). Compared with H-CHO mice, the H-CHO+MR diet markedly decreased TMA levels in cecal contents and colonic contents of mice, and notably decreased *CutC* and *CutD* expression levels as well as CutC activity both in mouse cecal and colonic contents (*p* < 0.01).

### 3.5. MR Reduced TMA-Production by Bacteria Escherichia fergusonii and Anaerococcus hydrogenalis

**Figure 6:**
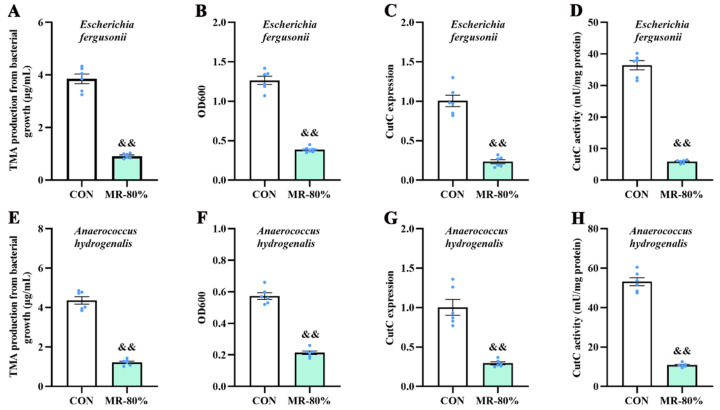
Effects of MR on TMA production (**A**,**E**), bacteria abundance (**B**,**F**), and CutC expression (**C**,**G**) and activity (**D**,**H**) in *Escherichia fergusonii ATCC 35,469* and *Anaerococcus hydrogenalis DSM 7454* cultured anaerobically in medium. MR, methionine restriction; TMA, trimethylamine; CutC, choline trimethylamine-lyase; CON, normal diet group; MR-80%, 0.17% methionine. ^&&^ *p* < 0.01 (MR-80% vs. CON).

*Escherichia fergusonii ATCC 35469* and *Anaerococcus hydrogenalis DSM 7454* were cultured in vitro to evaluate the inhibitory mechanism of MR regarding TMA production. Compared with the CON group, MR-80% markedly reduced TMA production by the bacteria *Escherichia fergusonii* and *Anaerococcus hydrogenalis* (Figure 6A,E), inhibited the growth of these two kinds of bacteria (Figure 6B,F), and significantly down-regulated *CutC* expression (Figure 6C,G) and CutC activity (Figure 6D,H) in *Escherichia fergusonii ATCC 35469* and *Anaerococcus hydrogenalis DSM 7454* (*p* < 0.01).

### 3.6. MR Reduced Inflammatory Response in Aorta Tissue of H-CHO-Diet-Fed Mice

**Figure 7:**
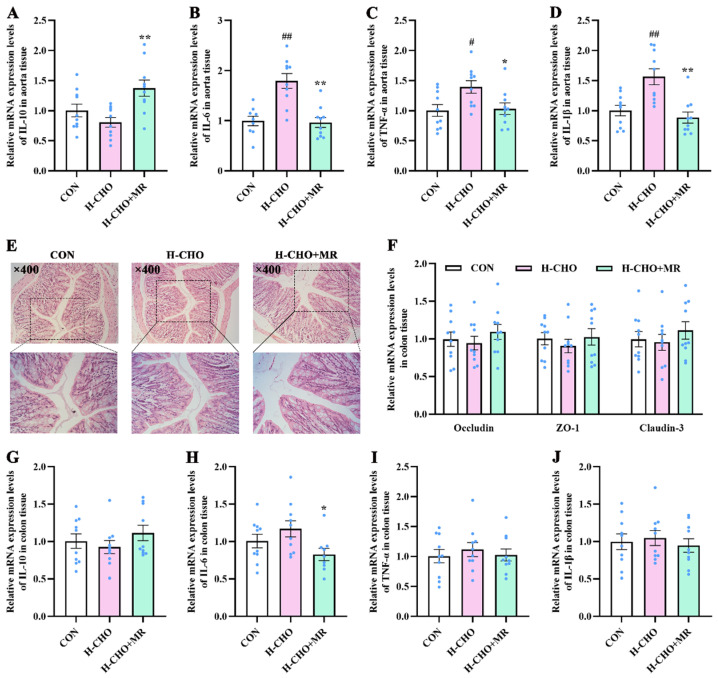
Effects of MR on mRNA expression levels of *IL-10* (**A**,**G**), *IL-6* (**B**,**H**), *TNF-α* (**C**,**I**), and *IL-1β* (**D**,**J**) in the aorta tissue and colon tissue, respectively, and on the morphological structure of the colon ((**E**), 400× magnification), as well as mRNA expression of *Occludin*, *ZO-1*, *Claudin-3* (**F**) in the colon of H-CHO mice. MR, methionine restriction; CON, normal diet group; H-CHO, high-choline diet group; H-CHO+MR, high-choline + methionine restricted diet group. ^#^ *p* < 0.05, ^##^ *p* < 0.01 (H-CHO vs. CON); * *p* < 0.05, ** *p* < 0.01 (H-CHO+MR vs. H-CHO).

Compared with CON mice, the H-CHO diet notably up-regulated the mRNA expression of IL-6 (Figure 7B), TNF-α (Figure 7C), and IL-1β (Figure 7D) levels in aorta tissue of mice (*p* < 0.05). Compared with H-CHO mice, the H-CHO-MR diet notably up-regulated the mRNA expression of IL-10 (Figure 7A), markedly down-regulated the mRNA expression of IL-6, TNF-α, and IL-1β levels in the aorta tissue of mice, and significantly decreased the mRNA expression levels of IL-6 in the colon tissue (Figure 7H) of mice (*p* < 0.05). Moreover, no morphological changes were observed in mouse colon tissue among the CON, H-CHO, and H-CHO+MR groups (Figure 7E). Furthermore, no significant difference was observed in mRNA expression of Occludin, zonula occludens-1 (ZO-1), and Claudin-3 (Figure 7F) in the colon tissue of mice among the three groups (*p* > 0.05).

### 3.7. MR Increased SCFA Levels in H-CHO-Diet-Fed Mice

The in vivo experiment revealed that compared with CON mice, the H-CHO diet significantly decreased acetate (Figure 7A), propionate (Figure 7B), butyrate (Figure 7C), and total acid (Figure 7D) levels in mouse cecal contents (*p* < 0.05). Compared with mice in the H-CHO group, the H-CHO+MR diet significantly elevated acetate, propionate, butyrate, and total acid levels in mouse cecal contents (*p* < 0.05).

### 3.8. Sodium Butyrate Supplementation Decreased TMA Production by Bacteria Escherichia fergusonii and Anaerococcus hydrogenalis

**Figure 8:**
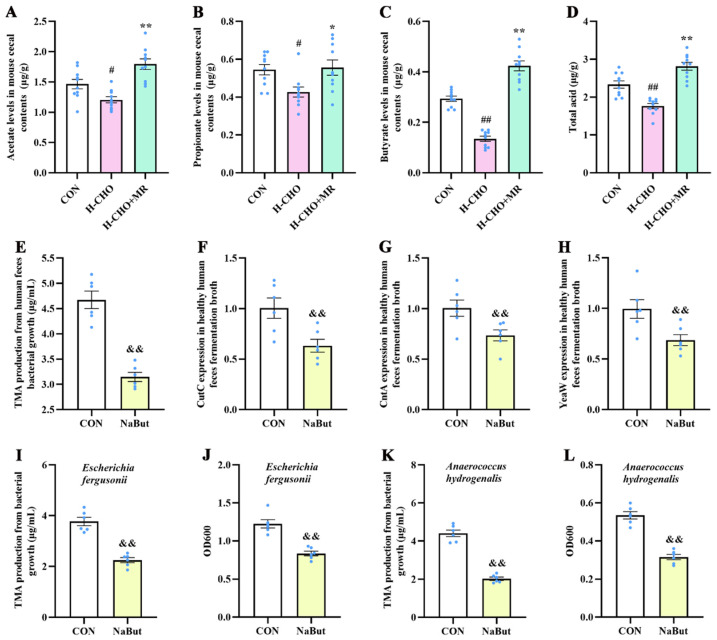
Effects of MR on acetate (**A**), propionate (**B**), butyrate (**C**), and total acid (**D**) levels in the cecal contents of mice fed with the H-CHO diet, and the effects of sodium butyrate supplementation on TMA production by bacteria from human feces (**E**) cultured anaerobically in medium, *CutC*, *CntA*, and *YeaW* expression in healthy human fecal fermentation broth (**F**–**H**), TMA production from *Escherichia fergusonii ATCC 35469* and *Anaerococcus hydrogenalis DSM 7454* (**I**,**K**), and bacteria abundance of *Escherichia fergusonii ATCC 35469* and *Anaerococcus hydrogenalis DSM 7454* (**J**,**L**). MR, methionine restriction; TMA, trimethylamine; CutC, CntA, and YeaW, trimethylamine-lyase; NaBut, sodium butyrate; CON, normal diet group; H-CHO, high-choline diet group; H-CHO+MR, high-choline + methionine restricted diet group. ^#^ *p* < 0.05, ^##^ *p* < 0.01 (H-CHO vs. CON); * *p* < 0.05, ** *p* < 0.01 (H-CHO+MR vs. H-CHO); ^&&^ *p* < 0.01 (NaBut vs. CON).

To assess the effects of SCFA (especially butyrate) on TMA production, sodium butyrate was added to human fecal fermentation broth and bacterial media, and then the TMA levels and bacterial abundance were determined. In the in vitro experiment, compared with the CON group, sodium butyrate supplementation significantly decreased TMA production from bacterial growth, including human fecal bacteria (Figure 7E), *Escherichia fergusonii ATCC 35469* (Figure 7I), and *Anaerococcus hydrogenalis DSM 7454* (Figure 7K), significantly decreased bacterial abundance (Figure 7J,L), and significantly down-regulated *CutC*, *CntA*, and *YeaW* expression levels in human fecal fermentation broth (Figure 8F–H) (*p* < 0.01).

## 4. Discussion

In this study, we focused on the gut microbiota-driven TMA/FMO3/TMAO pathway and evaluated the inhibitory effects of MR on TMA production by combining in vitro and in vivo experiments, circulating TMAO levels, and gut microbiota composition. Our results support the hypothesis that MR alleviates choline-induced TMAO elevation by manipulating gut microbiota in mice.

MR decreased TMA and TMAO levels in H-CHO-diet-fed mice. We initially tested the influences of different levels of MR on TMA production in an in vitro study. Our results revealed that TMA levels were significantly reduced by MR-60% and MR-80%, and MR-80% is more effective. MR-80% highly significantly decreased TMA levels and expression levels of TMA-lyases including *CutC*/*D*, *CntA*/*B*, and *YeaW*/*X* in mouse colonic contents and the human feces fermentation broth, indicating the inhibitory effects of MR on TMA production. An increased intake of dietary TMA precursors was reported to notably elevate the level of circulating TMAO [7,43], whereas MR can reduce plasma TMA and TMAO levels in both HFD- and normal-diet-fed mice [26,27]. To further evaluate the mechanism of MR on decreasing plasma TMA and TMAO levels, we carried out the in vivo experiment. The results revealed that MR reduced plasma TMA and TMAO levels in H-CHO-diet-fed mice. Gut microbiotas degrade dietary choline, betaine, and L-carnitine into TMA, which is further transformed into TMAO in liver through the action of FMO3 [1,44,45]. The increase in hepatic *FMO3* expression promoted the synthesis of TMAO from dietary precursors [46]. However, no significance was observed among the three groups in our present study and indicated that hepatic FMO3 may not be a predominant plasma TMAO inhibitory mechanism for our model, namely, MR mediated the decrease in plasma TMAO levels is not by decreasing the hepatic expression of FMO3. Previous researches in mice and humans suggested that TMAO production was dependent on variability of the gut microbiota species [6,47]. Of note MR was reported to improve gut microbiota composition in normal, HFD, and aging mice [28,29,40,48]. Therefore, MR may decrease plasma TMAO levels not by altering the hepatic expression of FMO3 but through decreasing intestinal TMA production via manipulating the gut microbiota composition.

MR improved the gut microbiota composition in H-CHO-diet-fed mice. Our data revealed that MR notably increased the Shannon index in mice, indicating an alteration in gut microbiota composition. *Escherichia-Shigella* and *Anaerococcus*, can metabolize choline to TMA through their own CutC and CutD and identified as TMA-producing bacteria [14,49,50,51], were significantly increased in H-CHO-diet-fed mice, indicating higher levels of TMAO in mice. On the contrary, despite the intake of the H-CHO diet, MR intervention notably reduced the abundance of *Escherichia-Shigella* and *Anaerococcus*, and notably reduced intestinal TMA levels in mice. Consistent with the significantly decreased abundance of TMA-producing bacteria, the H-CHO+MR diet significantly decreased *CutC/D* expression and CutC activity as well as TMA levels in mouse cecal/colonic contents. Subsequently, of the identified two kinds of reported TMA-producing human commensal strains including *Escherichia fergusonii* and *Anaerococcus hydrogenalis* [49] were cultured under anaerobic condition in medium to further evaluate the inhibitory effects of MR on TMA production. The results showed that MR-80% significantly inhibited bacterial growth, *CutC* expression levels, and CutC activity, thus significantly decreased TMA production from bacterial growth. The combined data revealed that MR significantly decreased the abundance of TMA-producing bacteria in the intestine, and thus decreased intestinal TMA level. TMA can enter the circulation through the intestinal barrier [52], and the reduced plasma TMA level is consistent with this contention. However, we previously found that the MR diet significantly increased TMA levels in colonic contents of mice under HFD [24], accompanied by decreased plasma TMA levels [26]. One potential explanation for these results is that MR improved long-term HFD-induced intestinal barrier dysfunction and relatively reduced the permeability of the intestine to TMA (consistent with normal group levels) [53]. In the current study, MR did not change the barrier function of colon in mice fed with the H-CHO diet and had no effect on the absorption of intestinal TMA. Therefore, MR intervention in this study consistently decreased TMA levels in the intestine and the plasma. In addition, *Bifidobacterium* is a genus of bacteria that has been extensively reported to be negatively correlated with circulating TMAO concentrations in humans [54,55] and mice [12]. A gut microbiota study revealed that *[Eubacterium]_coprostanoligenes_group* exhibits positive relation with TMAO levels, and through further analysis, the authors found that *Eubacterium* is a potential TMA producer [56]. *Oscillibacter* is another gut microbial species that positively correlates with plasma TMAO [13,57]. In the present study, the H-CHO-MR diet notably elevated the abundance of *Bifidobacterium*, and markedly reduced the abundance of *Eubacterium* and *Oscillibacter*. Our data indicate that circulating TMAO levels are correlated with specific gut microbiota profiles that might be involved in the regulation of plasma TMAO levels, which is in line with previous studies [6,47]. Taken together, these results showed that MR improved gut microbiota composition and decreased plasma TMAO levels by reducing intestinal TMA production via inhibiting TMA-producing bacteria.

SCFA are also important contributors to the MR-mediated decrease in TMAO by inhibiting TMA-producing bacteria. SCFAs, especially butyrate, were reported to reduce TMA production [32,58]. MR is capable of increasing SCFA-producing bacteria while decreasing the abundance of *Escherichia-Shigella* in mice fed with low-, medium-, or high-fat diets [24,29]. Consistent with these studies, MR markedly enhanced the abundance of SCFA-producing bacteria in this study. The genus *Bacteriodes* is a kind of SCFA-producing bacteria from the *Bacteroidetes* phylum [59], the increased abundance of which might contribute to the anti-atherosclerotic effect [60]. In our study, the H-CHO diet markedly reduced the abundance of *Bacteriodes*, which is markedly enhanced by the H-CHO+MR diet. Other SCFA-producing bacteria including *Lachnospiraceae NK4A136-group* [61], *Bifidobacterium* [24], *Faecalibaculum* [62], and *Roseburia* [63], all of which were notably increased in H-CHO+MR diet-fed mice and may exert inhibitory effects on TMA-producing bacteria including *Escherichia fergusonii* and *Anaerococcus hydrogenalis* [64]. Conversely, H-CHO mice showed markedly reduced abundance of *Bacteriodes*, *Bifidobacterium*, *Lachnospiraceae NK4A136-group*, and *Ruminococcaceae_UCG-014* [62]. It is noteworthy that previous studies found the abundance of butyrate-producing bacteria was inversely related with the abundance of *Escherichia-Shigella*, and suggest that butyrate may have an inhibitory relationship with *Escherichia-Shigella* [64]. In the present study, the H-CHO diet significantly decreased SCFAs (including butyrate, propionate, and acetate) levels and total acid in cecal contents of mice, whereas MR significantly increased these indicators. Of note, sodium butyrate supplement markedly reduced the expression of *CutC*, *CntA*, and *YeaW* as well as decreased TMA production from bacterial growth in human feces fermentation broth. Furthermore, sodium butyrate treatment inhibited the growth of *Escherichia fergusonii* and *Anaerococcus hydrogenalis*, thus inhibiting TMA production by bacteria. Being identified as the main energy source for the intestines [65], butyrate plays important roles in lipid and glucose metabolism therefore give rise to energy expenditure [66,67,68]. Raised butyrate consequent to microbiota changes is usually associated with reduced body weight [29,69]. Consistent with this notion, our results revealed that the H-CHO+MR diet notably elevated butyrate levels and markedly reduced body weight and body weight gain in mice. These data indicate that elevated butyrate may be one of the mechanisms of weight loss and lipid reduction under MR [70,71]. Taken together, these data implied that MR promoted the abundance of SCFA-producing bacteria and enhanced SCFAs production decreased TMA production by inhibiting bacteria growth and the expression as well as the activity of TMA-lyases.

TMAO has been reported to promote vascular inflammation [72], which is now being pinpointed as a pivotal regulatory process in the development of AS [73,74], and diversity in gut microbiota and the presence of particular species was reported to be associated with inflammation [75]. *Helicobacter* is a very prevalent bacterial gastroduodenal pathogen, the chronic colonization of which exacerbates inflammatory response [76,77], and the serum TMAO levels were reported to synergize with the pro-inflammatory effects of *Helicobacter* [78]. Of note, *Helicobacter* has been positively correlated both epidemiologically and pathogenetically with AS, and chronic *Helicobacter* infection was related with a higher risk of stroke [79]. According to a published study, improved HFD-induced AS was associated with decreased abundance of *Helicobacter* [60]. Aside from being a type of TMA-producing bacterium, *Escherichia-Shigella* is also a pro-inflammatory bacterium [80], whose abundance was found to be positively associated with circulating TMAO levels [81]. *Candidatus Saccharimonas* belongs to the superphylum *Patescibacteria*, which is associated with inflammatory disease [82]. In this study, the H-CHO+MR diet significantly decreased the abundance of *Helicobacter*, *Escherichia-Shigella*, and *Candidatus Saccharimonas* in mice. Corresponding with the variations in bacterial abundance, the H-CHO+MR diet significantly down-regulated the mRNA expression of pro-inflammatory cytokines including *IL-6*, *TNF-α*, and *IL-1β* while significantly up-regulated the mRNA expression of anti-inflammatory cytokine *IL-10* in mice. Moreover, MR notably decreased the AS index. The combined data indicate that reduced inflammation in mice of the H-CHO+MR group was associated with decreased circulating TMAO levels and led to a lower risk of AS progression. It is of great importance that the methionine content of various foods is different; in general, animal-origin foodstuffs contain far more methionine than plant-derived foodstuffs [83] and the animal proteins contain higher methionine than those of vegetable proteins [84]. MR can be achieved through several ways: (1) simple dietary adjustments and choosing foods low in methionine such as quinoa, sorghum, lupin, and freshwater fish, etc. [85]; (2) diet formulations based on monomeric amino acids [86,87,88,89]; (3) oral intake or injection of methioninase [90,91]; (4) the screening and cultivating of low-methionine plant foods. In a proof-of-principle clinical study, middle-aged individuals subjected to a low methionine diet (about 2.92 mg kg^−1^ day^−1^, represented an 83% reduction in methionine intake) showed altered circulating metabolism, which further demonstrated therapeutic effects of MR on cancer [89]. Another similar clinical study also demonstrated that enteral dietary methionine restriction is safe and tolerable in adults with metastatic solid tumors and results in significant reduction in plasma methionine levels [86]. These studies verified the feasibility and effectiveness of MR diet, indicating the eventual appropriateness of MR in humans. Therefore, MR diet may be a promising dietary strategy for health improvement and disease treatment.

## 5. Conclusions

In conclusion, our results show that MR decreased TMA production in both in vitro and in vivo experiments, down-regulated *CutC*/*CutD* expression, inhibited TMA-producing bacteria including *Escherichia-Shigella* (*Proteobacteria* phylum) and *Anaerococcus* (*Firmicutes* phylum), increased SCFA-producing bacteria and SCFA levels, and reduced plasma TMAO levels. Furthermore, both MR and sodium butyrate supplementation inhibited TMA production from *Escherichia fergusonii ATCC 35469* and *Anaerococcus hydrogenalis DSM 7454* in the in vitro fermentation experiment. This finding suggests a novel mechanism of MR action by altering the gut microbiota-driven TMA/FMO3/TMAO pathway, which involves manipulating the gut microbiota independently of hepatic gene expression and enzyme activity of FMO3. Our study also provides a scientific theoretical basis for designing an individualized low-methionine diet to reduce circulating TMAO levels and the development and application of low-methionine food for the prevention and treatment of AS.

## Data Availability

All data are contained within the article and Supplementary File.

## Supplementary Materials

The following supporting information can be downloaded at: https://www.mdpi.com/article/10.3390/nu15010206/s1, Table S1: The compositions of the experimental diets (g/100 g); Table S2: Primer sequences for qRT-PCR.
