# Supplementary material for: Dietary Methionine Restriction Alleviates Choline-Induced Tri-Methylamine-N-Oxide (TMAO) Elevation by Manipulating Gut Microbiota in Mice
Source: Nutrients. 2023 Jan 1;15(1):206. doi: 10.3390/nu15010206 (PMC9823801; doi:10.3390/nu15010206)
Supplement: Supplementary file 1 [file nutrients-15-00206-s001.zip › nutrients-1969177-supplementary.pdf]

Supporting information

Supplemental Tables

Table S1. The compositions of the experimental diets (g/100g)

Ingredient	CON	H-CHO	H-CHO+MR
Soy protein	7.59	7.59	7.59
L-Arginine	0.66	0.66	0.66
L-Histidine	0.18	0.18	0.18
L-Isoleucine	0.51	0.51	0.51
L-Leucine	0.64	0.64	0.64
L-Lysine	0.11	0.11	0.11
L-Methionine	0.69	0.69	0.00
L-Phenylalanine	0.85	0.85	0.85
L-Threonine	0.65	0.65	0.65
L-Tryptophan	0.10	0.10	0.10
L-Valine	0.57	0.57	0.57
L-Glutamic acid	1.37	1.37	2.06
L-Glycine	2.08	2.08	2.08
Corn starch	64.00	62.91	62.91
Maltodextrin	5.00	5.00	5.00
Sucrose	0.10	0.10	0.10
Soybean oil	2.00	2.00	2.00

Pork Lard	2.20	2.20	2.20
Cellulose	5.00	5.00	5.00
Mineral mixture-AIN-76A*	3.50	3.50	3.50
Mineral vitamin-AIN-76A*	1.00	1.00	1.00
Choline chloride	0.20	1.20	1.20
CMC	1.00	1.00	1.00
Total	100.00	100.00	100.00

Amino acid composition of soy protein was as follows: 6.19% leucine, 4.11% isoleucine, 5.49% valine, 1.18% methionine, 1.66% cysteine, 4.09% phenylalanine, 2.57% tyrosine, 4.83% lysine, 2.21% threonine, 1.07% tryptophan, 1.99% histidine, 6.11% arginine, 3.30% serine, 3.25% alanine, 5.56% proline, 3.27% glycine, 17.49% glutamic acid 9.44% aspartic acid. 1 g cysteine is equal to 0.64 g methionine.

Table S2. Primer sequences for qRT-PCR

Primer Name Sequence	Sequence
CutC-F	5'-TTYGCIGGITAYCARCCNTT-3'
CutC-R	5′-TGNGGYTCIACRCAICCCAT-3'
CutD-F	5'-GATTAACACCGCCGTCGAAA-3'
CutD-R	5'-TCCACCAGCCATTCGAGATT-3'
CntA-F	5'-TAYCAYGCITGGRCITTYAARCT-3'
CntA-R	5'-RCAGTGRTARCAYTCSAKRTAGTTRTCRAC-3'
CntB-F	5'-GTTTATCGCRGGCGGTATTGGTAT-3'

CntB-R	5'-GCCTGNGGATGCTGRACTA-3'
YeaW-F	5'-GCCTGGGCGCGAAAGTGCT-3'
YeaW-R	5'-TACNGAGTCGGAGAAANNTGGATG-3'
YeaX-F	5'-AGTTCGCNGCATNACACCTC-3'
YeaX-R	5'-AATACCGCCCGCGATAAACAGATG-3'
16S-F	5'-GTGCCAGCMGCCGCGG-3'
16S-R	5'-GGACTACNNGGGTATCTAAT-3'
FMO3-F	5'-GGAACCAGGAATATGGAAG-3'
FMO3-R	5'-GGTGACCTTCTGAGCTACAT-3'
IL-10-F	5'-CATTCATGGCCTTGTAGACACC-3'
IL-10-R	5'-CTTAATGCAGGACTTAAGGGTTA-3'
IL-6-F	5'-ACACACTGGTTCTGAGGGAC-3'
IL-6-R	5'-TACCACAAGGTTGGCAGGTG-3'
IL-1β-F	5'-GAAATGCCACCTTTTGACAGTG-3'
IL-1β-R	5'-TGGATGCTCTCATCAGGACAG-3'
TNF-α-F	5'-CTGAACTTCGGGGTGATCGGT-3'
TNF-α-R	5'-TCCTCCACTTGGTGGTTTGCTAC-3'
Claudin-3-F	5'-ACCAACTGCGTACAAGACGAG-3'
Claudin-3-R	5'-CAGAGCCGCCAACAGGAAA-3'
Occludin-F	5'-ATGTCCGGCCGATGCTCTC-3'
Occludin-R	5'-TTTGGCTGCTCTTGGGTCTGTAT-3'
ZO-1-F	5'-ACCCGAAACTGATGCTGTGGATAG-3'

ZO-1-R	5'-AAATGGCCGGGCAGAACTTGTGTA-3'
β-actin-F	5'-GGGTCAGAAGGACTCCTATG-3'
β-actin-R	5'-GTAACAATGCCATGTTCAAT-3'

CutC, choline TMA-lyase; *CutD*, choline TMA-lyase activating protein; *CntA/B*, a two-subunit Rieske-type oxygenase/reductase; *YeaW/X*, another Rieske-type oxygenase/reductase; *FMO3*, flavin-containing mono-oxygenase 3; *IL-10*, interleukin 10; *IL-6*, interleukin 6; *TNF-α*, tumor necrosis factor alpha; *IL-1β*, interleukin 1 beta; *ZO-1*, zonula occludens-1.
